# Comparison of two attenuated infectious bursal disease vaccine strains focused on safety and antibody response in commercial broilers

**DOI:** 10.14202/vetworld.2021.70-77

**Published:** 2021-01-11

**Authors:** Thotsapol Thomrongsuwannakij, Nataya Charoenvisal, Niwat Chansiripornchai

**Affiliations:** 1 Akkhraratchakumari Veterinary College, Walailak University, Nakorn Si Thammarat 80160, Thailand; 2 Avian Health Research Unit, Department of Veterinary Medicine, Faculty of Veterinary Science, Chulalongkorn University, Bangkok 10330, Thailand

**Keywords:** broilers, immunosuppressive effects, infectious bursal disease, vaccination

## Abstract

**Background and Aim:**

Infectious bursal disease (IBD) or Gumboro disease is one of the most detrimental diseases in the poultry industry worldwide. Previous scientific studies have shown that live IBD vaccination might induce transient immunosuppression, leading to suboptimal vaccine responses, and therefore lack of protection against other infectious diseases; therefore, selecting an IBD vaccine in commercial farms is a concern. This study aims to compare two commercially attenuated IBD vaccines (intermediate and intermediate-plus strains) in terms of safety and antibody response to IBD and Newcastle disease viruses (NDV) in commercial broilers.

**Materials and Methods:**

Overall, 216 Cobb broiler chickens were divided into three groups based on the IBD vaccine strain administered: V217 strain (Group 1), M.B. strain (Group 2), and an unvaccinated group (Group 3). Groups 1 and 2 were orally vaccinated with Hitchner B1 NDV vaccine strain 7 days after IBD vaccination. Blood samples were collected at IBD vaccination day (15 days of age) and at 7, 14, 21, and 28 days post-IBD vaccination. The immunosuppressive effects of the IBD vaccination were determined by NDV antibody response, the bursa:body weight (B:BW) ratio, and the histopathological lesion scores of the bursa of Fabricius. Phylogenetic analysis was also performed.

**Results:**

Phylogenetic analysis revealed that the M.B. strain belonged to a very virulent IBD strain, whereas the V217 strain belonged to a classical IBD virus strain. NDV antibody titers of the two vaccinated groups increased after ND vaccination, reaching their maximum at 14 days post-ND vaccination and decreasing thereafter. The V217 group presented the highest NDV humoral response from 7 days post-vaccination (dpv) to the end of the study. The mean NDV antibody titer of the V217 group was significantly (p<0.05) higher than that of the M.B. group at 14 dpv. In addition, the V217 strain-induced lower bursal lesions post-IBD vaccination and a higher B: BW ratio at 7 and 21 dpv compared to the M.B. group. The higher B: BW ratio, lower bursal lesions, and higher ND antibody response present in the V217 group indicate that the V217 strain induces lower immunosuppressive effects compared to the M.B. strain.

**Conclusion:**

The results of this study indicate that IBD vaccine selection merits consideration, as avoiding the immunosuppressive effects induced by live IBD vaccination and the consequent impact on response to other vaccines is important.

## Introduction

Infectious bursal disease (IBD), also known as Gumboro disease, is one of the most widespread immunosuppressive avian diseases, causing high morbidity and mortality in commercial broilers, and even up to 100% mortality in susceptible white leghorn chickens [1,2]. This disease is caused by the IBD virus (IBDV), which is a single-shelled, non-enveloped virus with a double-stranded ribonucleic acid (RNA) genome [2,3]. This virus is considerably resistant to harsh environments due to the absence of an envelope. IBD can cause immunosuppression in young chickens due to B lymphocyte depletion [4]. Furthermore, it has been reported that macrophages and monocytes may be susceptible to IBDV infection [4,5]. Macrophages have been thought to serve as IBDV carriers from the infection site in the gut to the bursa of Fabricius and other peripheral organs [5-7].

Immunosuppression decreases the resistance of these birds to other infections and also leads to an inadequate immune response to vaccination [8]. Immunosuppression following IBDV infection likely causes substantial economic loss due to vaccination failure, subsequent susceptibility to opportunistic pathogens, and loss of production [2]. The characteristic clinical signs of diseased chickens are dehydration and petechial hemorrhage at the thigh and breast muscles or at the junction between the proventriculus and the gizzard. The bursa of Fabricius of infected birds presents with inflammation, hemorrhage, or atrophy, depending on the infection period [9]. Vaccination plays an essential role in successfully controlling IBD [10,11]. At present, IBD vaccines are commercially available, including live attenuated, killed, immune complex, and vector vaccines [2]. Live attenuated vaccines, which are the most prevalently used IBD vaccines in the field, can be categorized into three groups: Mild, intermediate, and intermediate-plus or hot vaccines [12]. The viruses in mild vaccines exhibit poor efficacy in the presence of certain levels of maternally derived antibodies (MDAs) [13] and against very virulent IBDV (vvIBDV). In contrast, the intermediate and intermediate-plus or hot vaccines have much better efficacy and may break through the high level of MDAs and induce antibodies against IBDV. However, the various attenuation levels of commercially live IBD vaccines result in varying immunosuppression levels, increasing the birds’ vulnerability to infection by other pathogens [14-16]. In addition, an efficient vaccination program depends on the time of vaccination, which can be affected by residual MDA levels [17]. Consequently, the safety and efficacy of these vaccine types still remain an essential concern. In commercial broiler breeder farms, killed IBDV vaccines have been routinely used to achieve a high antibody response against IBDV and consequently transmit the IBDV antibodies to the offspring, which may interfere with the immune response to vaccines [17,18], an issue that farmers should be aware of before selecting an IBDV vaccine. In Thailand, similar to other countries, broiler producers tend to use the most virulent IBDV vaccines available, even if clinical Gumboro disease has not been commonly diagnosed in the previous broiler cycles.

This study aims to compare two commercially attenuated IBD vaccines in terms of safety, antibody response to the IBD vaccine, and immunosuppressive effect using the Newcastle disease virus (NDV) vaccination model in commercial broilers.

## Materials and Methods

### Ethical approval

The guidelines and legislative regulations on the use of animals for scientific purposes of Chulalongkorn University, Bangkok, Thailand, were followed as is certified in permission number 1931005.

### Study period and location

This research was conducted for 9 months (January to September 2019), consisting of pre-research, experimental, and laboratory examination. The birds were reared in the experimental facilities, Faculty of Veterinary Science, Chulalongkorn University. The laboratory examinations were conducted at the Department of Veterinary Medicine, Chulalongkorn University.

### Animals

A total of 216 female, unvaccinated, 1-day-old Cobb-500 broiler chicks were obtained from the same broiler breeder flock and hatchery. Breeder chickens were vaccinated with attenuated and inactivated IBDV vaccines as follows: At 14 days, Bursine 2 (Zoetis, USA); at 24 days, IBD Blen (Boehringer Ingelheim, Germany); and at 18 weeks, Provac 4 (Zoetis, USA). The chicks were maintained in three separate isolation units and fed *ad libitum* on commercial poultry feed (Betagro, Bangkok, Thailand).

### Experimental design

A total of 216 female broilers were divided into three groups of 72 chicks each. Each group was divided into four replicates of 18 chicks each. In Group 1, broilers were vaccinated with intermediate-plus vaccine strain V217, one dose per bird orally administered at 15 days old, which is an optimal day for vaccination according to the Deventer formula. The breakthrough titer was 636 enzyme-linked immunosorbent assay (ELISA) units, with 75% of the flock being susceptible [19]. Each dose of vaccine contained approximately 10^1.5^-10^3^ ELD_50_ of IBDV. Seven days after IBDV vaccination, the chickens were vaccinated with one dose of the live ND vaccine (Hitchner B1 strain) by eye drop. In Group 2, broilers were vaccinated with the intermediate M.B. strain, one dose per bird orally administered at 15 days old, again according to the Deventer formula. The breakthrough titer was also 636 ELISA units with 75% of the flock being susceptible [19]. Seven days after IBD vaccination, the chickens were vaccinated with one dose of the live ND vaccine (Hitchner B1 strain) by eye drop. Group 3 was a negative control group, and the broilers did not receive any vaccine. At 15, 29, and 43 days old, all birds were weighed; feed intake was recorded, and feed conversion ratio (FCR) was calculated by the amount of feed consumed divided by the amount of weight gain in a period of time.

### Humoral immune response

Specific antibody titers to IBDV and NDV were analyzed in serum samples using ELISA for IBDV strain D78 (BioChek, USA) and by a hemagglutination inhibition (HI) test, respectively. To collect hygienic samples, 20 birds per group were bled at 1, 7, 15 (IBD vaccination day), 22, 29, 36, and 43 days old. A summary of sample collection in each group is presented in Table-1.

**Table-1:** Sample collections in each group.

Sampling schedule	Number of blood samples/house	Serological analysis	Number of bursa of Fabricius
1 day of age	20	IBD, ND	0
7 days of age	20	IBD, ND	0
Vaccination day, 15 days of age	20	IBD, ND	12 (4 from each group)
7 dpv, 22 days of age	20	IBD, ND	12 (4 from each group)
14 dpv, 29 days of age	20	IBD, ND	12 (4 from each group)
21 dpv, 36 days of age	20	IBD, ND	12 (4 from each group)
28 dpv, 43 days of age	20	IBD, ND	12

dpv=days post-vaccination, IBD=Infectious bursal disease, ND=Newcastle disease

### Bursa:body weight (B:BW) ratio, B:BW index, and bursa scoring

On vaccination day, and 7, 14, 21, and 28 days post-vaccination (dpv), one bird in each replicate was euthanized, and the bursa of Fabricius was collected to measure B:BW ratio (Table-1) and histopathological lesion score (HLS) determination. The B:BW ratio was calculated by the bursa of Fabricius weight (g)/BW (g) × 1000. The B:BW index was calculated by B:BW ratio of vaccinated birds/B:BW ratio of the control group. HLS was determined according to Muskett *et al*. [20] using the following scale: (0) No damage; (1) mild necrosis in isolated follicles; (2) moderate generalized lymphocyte depletion or isolated follicles, with severe depletion; (3) over 50% of follicles with severe lymphocyte depletion; (4) outline of follicles only remaining with few lymphocytes and increase in connective tissue, cysts, and thickened corrugated epithelium; and (5) loss of all follicular architecture with fibroplasia.

### RNA extraction and reverse transcription-polymerase chain reaction (RT-PCR)

At 43 days old, the bursae of Fabricius of six birds per group were collected, and RT-PCR was performed to detect IBDV. Furthermore, RNA virus was extracted from vaccines used in Groups 1 and 2. RNA from bursa tissue samples and vaccines was extracted using a commercial kit (NucleoSpin Extract Viral RNA Kit, Macherey-Nagel, Germany), as described by the manufacturer. The RT-PCR assay was conducted using a primer pair that amplifies a 743-bp region of vVP2 as previously described [21]. The RT-PCR products were analyzed using 1.2% agarose gel electrophoresis and visualized under an ultraviolet transilluminator.

### Nucleotide sequence analysis and phylogenetic tree construction

The RT-PCR products of the vaccines from both groups were purified using a commercial kit (NucleoSpin Gel and PCR Clean-Up, Macherey-Nagel, Germany) and were submitted for nucleotide sequencing to First Base Laboratories (Seri Kembangan, Selangor, Malaysia) with specific primer sets. Basic Local Alignment Search Tool analysis was carried out on the website of the National Center for Biotechnology Information. Sequence analysis was performed using the MEGA 5.1 program [22]. The nucleotide sequences of two IBD vaccines used in this study, as well as very virulent, variant, classic, and serotype strains, were included. The genome sequences used as reference strains were taken from GenBank. The accession numbers included (1) very virulent strain: D49706 for OKYM, AF092943 for HK46, DQ916217 for Singapore97S182, AJ318897 for UK661, EF517528 for Harbin-1, DQ916248 for Thailand01_TH4, DQ916247 for Thailand01_TH3, GQ451330 for HLJ-0504, MW248904 for vaccine strain M.B., and AJ586962 for MB_VP2, (2) variant strain: AF281238 for strain T1, DQ916213 for Mexico04M92, AF133904 for strain E, and DQ916181 for Colombia01C10, (3) classic strain: AF362747 for Cu-1wt, AJ586961 for Bursine plus, MW248903 for vaccine strain V217, DQ916252 for Thailand97_TH4, MW248905 for Thai4 Classic, AJ586966 for Nobilis 228E, and AY332560 for IBD Blen, and (4) Serotype 2: U30818 for OH, and AF362773 for 23/82. The phylogenetic tree was constructed using the Maximum Likelihood algorithm in the MEGA X program.

### Statistical analysis

Antibody titers against IBDV and NDV, BW, and bursa weight were compared between groups using one-way analysis of variance and Duncan’s multiple range tests. Differences between B:BW ratios and HLS were calculated using Chi-square and Kruskal–Wallis tests. Differences between groups were considered significant at p<0.05. Statistical analysis was performed using Statistical Package for the Social Sciences for Windows v. 22 (IBM Corp., NY, USA).

## Results

### Antibody titers against IBDV and NDV

Maternal IBD antibody titers gradually decreased as broilers aged. At 1 and 7 days old, the broilers had not been vaccinated yet, so IBD and ND antibody titers were represented in all broiler groups. At 15 days old, the IBD vaccination date, there was no statistically significant difference in the IBD antibody titers of all groups. After vaccination, the IBD antibody titers in the vaccinated groups increased compared to the non-vaccinated group. At 36 and 43 days old, birds of Group 2 (the M.B. vaccinated group) displayed the highest IBD antibody titers (Group 1, 6099.1 ± 4174.97; and Group 2, 8677.95 ± 3391.68; p<0.05) among all groups (Table-2). ND vaccines were administered at 22 days of age. At 36 and 43 days old, the ND antibody titers of birds in Group 1 displayed the highest ND antibody titers (30.85±31.74 and 21.85±28.94, respectively) compared to Groups 2 and 3 (Table-3).

**Table-2:** Antibody titers against IBD by enzyme-linked immunosorbent assay and Newcastle disease by hemagglutination inhibition test.

Group	IBD antibody titers at different ages (days) (mean±standard deviation)						

	1	7	15	22	29	36	43
1 (V217)			363.35±252.82	351.6±441.94	932±1988.15	1294.05±2094.63^a^	5232.15±4115.59^a^
2 (M.B.)	6162.2±2442.51	1576.95±502.53	350.35±158.27	241.35±110.08	1474.9±2867.48	6099.1±4174.97^b^	8677.95±3391.68^b^
3 (control)			403.8±152.96	264.65±168.23	117.55±47.71	116.65±73.77^c^	112.55±66.62^c^

Different superscripts in each column mean statistically significant difference (p<0.05), IBD=Infectious bursal disease

**Table-3:** Antibody titers against ND by hemagglutination inhibition test.

Group	ND antibody titers at different ages (days) (mean±standard deviation)						

	1	7	15	22	29	36	43
1 (V217)			2.95±1.67	2.00±1.12	1.15±0.67	30.85±31.74^a^	21.85±28.94^a^
2 (M.B.)	124.80±83.64	16.40±6.07	3.30±1.49	2.05±0.94	1.10±0.31	15.55±9.84^b^	17.30±15.63^a,b^
3 (control)			3.20±1.51	2.50±1.32	1.20±0.41	1.00±0.00^c^	1.00±0.00^c^

Different superscripts in each column mean statistically significant difference (p<0.05). ND=Newcastle disease

### BW and feed conversion ratio

At 43 days old, broilers in Group 2 had the highest average BW (1.94±0.41 kg), while the FCR of broilers in Group 1 between 29 and 43 days was the lowest (2.30±0.45) among the three experimental groups. However, differences in BW and FCR of broilers at 15, 29, and 43 days old were not statistically significant (Table-4).

**Table-4:** Body weight (kilogram) and FCR.

Group	Body weight (mean±SD)			FCR (mean±SD)		
	
	15 day	29 day	43 day	1-15 day	15-29 day	29-43 day
1 (V217)	0.56±0.04	1.39±0.16	1.86±0.45	1.20±0.03	0.89±0.06	2.30±0.45
2 (M.B.)	0.56±0.03	1.43±0.09	1.94±0.41	1.17±0.03	0.94±0.00	2.40±0.2
3 (control)	0.57±0.06	1.43±0.07	1.93±0.41	1.17±0.01	0.93±0.04	2.36±0.48

FCR=Feed conversion ratio, SD=Standard deviation

### B:BW ratio, B:BW index, HLS, and RT-PCR of IBDV

B:BW ratios at 22 days of age were higher than B:BW ratios at 15 days of age; after 22 days, B:BW ratios continuously decreased until 43 days old. At 29 days old, or 14 dpv, birds in the vaccinated Group 1 showed significantly lower B:BW ratios (0.80±0.21) than the control group (Group 3, 1.23±0.25; p<0.05). Furthermore, at 43 days old, or 28 dpv, birds in vaccinated Groups 1 and 2 showed significantly lower B:BW ratios (0.25±0.08 and 0.27±0.07, respectively) than the control group (Group 3, 0.65±0.34; p<0.05) (Table-5). Vaccinated chickens had B:BW values between 0.8 and 1.20 in Group 1 and values between 0.42 and 1.22 in Group 2. Broilers in IBD vaccinated groups displayed higher HLS of the bursa than the unvaccinated group. At 36 (21 dpv) and 43 (28 dpv) days old, broilers of group 2 had the highest bursa HLS among the three groups (4±2 and 4.5±1, respectively). Furthermore, the HLS of the bursa of Fabricius of the vaccinated groups was significantly lower than those of the control group at 14 and 28 dpv (Table-6). Five out of six samples were positive for IBDV in both vaccinated groups (Groups 1 and 2) by RT-PCR, while there were no positives for IBDV in the control group (Group 3) (Table-6).

**Table-5:** Bursa weight (gram), BW (gram), and B:BW ratios.

Group	15 days old				22 days old				29 days old			
		
	Bursa	BW	B:BW ratio	B:BW index	Bursa	BW	B:BW ratio	B:BW index	Bursa	BW	B:BW ratio	B:BW index
1 (V217)	0.88 ±0.16	572.5 ±35.71	1.53 ±0.23	1.13	1.8 ±0.56	886.5 ±151.19	2.02 ±0.51	1.20	1.1 ±0.30	1366.25 ±34.00	0.80 ±0.21^a^	0.65
2 (M.B.)	0.94 ±0.16	572.5 ±42.72	1.65 ±0.28	1.22	1.58 ±0.56	936.25 ±58.51	1.67 ±0.48	0.99	1.31 ±0.19	1413.75 ±24.96	0.93 ±0.12^,b^	0.76
3 (control)	0.75 ±0.10	557.5 ±2.89	1.35 ±0.18	n/a	1.51 ±0.78	836.25 ±226.99	1.69 ±0.68	n/a	1.81 ±0.40	1470.00 ±34.16	1.23 ±0.25^b^	n/a

**Group**	**36 days old**						**43 days old**					
	
	**Bursa**	**BW**			**B:BW ratio**	**B:BW index**	**Bursa**	**BW**			**B:BW ratio**	**B:BW index**

1 (V217)	1.32±0.81	1797.50±116.73			0.76±0.50	0.94	0.5±0.19	1991.25±186.16			0.25±0.08^a^	0.38
2 (M.B.)	1.01±0.79	1787.50±50.58			0.56±0.43	0.69	0.53±0.10	1990.00±213.42			0.27±0.07^a^	0.42
3 (control)	1.5±0.96	1851.25±86.25			0.81±0.54	n/a	1.4±0.69	2168.75±100.20			0.65±0.34^b^	n/a

Different superscripts in each column mean statistically significant difference (p<0.05). n/a=Not applicable, BW=Body weight, B:BW=Bursa:body weight

**Table-6:** The histopathological lesion scores (HLS) of the bursa of Fabricius and the number of RT-PCR positive.

Group	The HLS of the bursa of Fabricius (mean±SD) at each age (days old)					No. of RT-PCR positive (%)

	15	22	29	36	43	
1 (V217)	1.5±0.58	2±0.82	3.25±0.96^a^	2.75±1.5	3.75±0.5^a^	5/6 (83.33)
2 (M.B.)	1±0	2±0	3.25±2.22^a^	4±2	4.5±1^a^	5/6 (83.33)
3 (control)	0.75±0.96	1±0.82	1±0^b^	1.5±0.58	1.75±0.5^b^	0/6 (0)

Different superscripts in each column mean statistically significant difference (p<0.05)

### Partial sequences of IBDV

**Figure-1:**
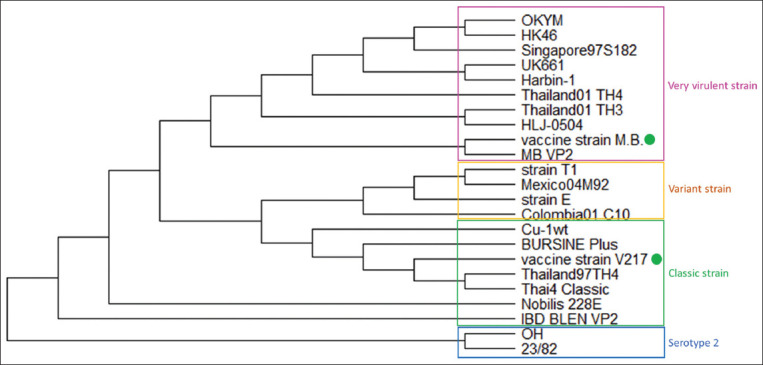
Phylogenetic analysis revealed that M.B. strain belongs to very virulent infectious bursal disease virus (IBDV) and V217 belongs to classic IBDV strain.

A partial sequence of the *VP2* indicated that virus from the vaccine strain V217 used in Group 1 was related to other classical IBDV strains, while the vaccine strain M.B. used in Group 2 was related to other vvIBDV strains. In addition, partial sequences of the *VP2* from Groups 1 and 2 were 93.23% similar. The phylogenetic results are shown in Figure-1.

## Discussion

IBD, also known as Gumboro disease, can cause morphologic and histological changes in the bursa of Fabricius. It can also cause significant economic losses due to the high mortality and high morbidity resulting from IBDV infection [2]. The immunosuppressive effect of IBDV vaccines is a concern due to the fact that the immunocompromised birds may not demonstrate sufficient titers of antibodies after vaccination against other diseases, such as NDV [4]. The IBDV vaccination has been used in the chicken industry worldwide to prevent IBDV infection. In Thailand, broiler breeders, broilers, and layers have been commonly vaccinated with the intermediate and/or intermediate-plus strains of IBD live and/or killed vaccines, depending on the type of chickens. Long-lived birds are also routinely vaccinated with killed IBDV vaccines to maintain a certain level of immune response to IBDV for prolonged protection. In this study, we compared two attenuated IBD vaccines (intermediate and intermediate-plus strains) in terms of safety, antibody response to IBD vaccine, and immunosuppressive effect using the NDV vaccination model in commercial broilers.

One-day-old chicks in both treatment groups came from the same hatchery and the same breed, and we randomly sampled their blood checking for IBDV antibody levels. Before each trial, a date for the IBD vaccination was calculated according to the Deventer formula [19] to reduce factors that may have influenced the results of the study. Interestingly, our study demonstrated that, although the IBD vaccine M.B. strain is commercially registered as an intermediate type, it was found that the M.B. vaccinated birds displayed significantly higher IBD antibody titers than the V217 vaccinated birds, which is rated as an intermediate-plus strain, at 14 and 21 dpv. Furthermore, the M.B.-vaccinated birds demonstrated significantly lower ND antibody titer by HI test at 14 dpv.

Rautenschlein *et al*. [23] reported that the intermediate-plus IBDV vaccine increased transient suppression of NDV antibody titer after NDV vaccination in commercial broilers, whereas a permanent suppression of ND antibody titer was observed in SPF layers. Although IBD is of great economic significance in the broiler industry and many studies have been conducted to determine the efficacy and immunosuppressive effects in SPF chickens [14,24-26], few studies have been performed in commercial broilers with residual MDAs [23,27-29].

Before IBDV live vaccines are put on the ­markets, most IBDV vaccines are evaluated for their immunosuppressive effects in SPF layer-type chickens to ­categorize the IBD vaccine as mild, intermediate, or intermediate-plus type [25,26], which may cause different results when compared to IBDV-vaccinated commercial broilers with residual MDAs. In addition, Lazarus *et al*. [30] described that the IBDV M.B. live vaccine is an intermediate to intermediate-plus strain originating from a vvIBDV, in agreement with our phylogenetic results, which showed that the IBDV vaccine M.B. strain was grouped in a cluster of vvIBDV, while the IBDV vaccine strain V217 was grouped in a cluster of classical strains of IBDV. This may be the reason why M.B. vaccinated birds showed a higher IBD antibody titer and greater immunosuppressive effect than the group that received the V217 strain.

BW and FCR of both treatment and control groups were not statistically significantly different. We found that the B:BW ratio of both treatment groups was markedly decreased at 14 dpv, and B:BW of treatment groups was significantly lower than that of the control group, similar to a previous study [31]. Likewise, the HLS of the bursa of Fabricius of treatment groups was significantly lower than that of the control group at 14 and 28 dpv.

To confirm that the IBDV vaccine in both groups could invade the bursal follicles, RT-PCR was performed to verify whether the follicles were positive for IBDV. The results indicated that 83.33% of the tested bursae were positive for IBDV, indicating that the broilers received proper IBDV vaccination in both treatment groups. In this study, we found that both IBDV vaccines could be present in the bursae of Fabricius at 28 dpv, which is in agreement with the study of Iván *et al*. [32], so broiler farmers may be made aware that IBDV can be detected as a result of vaccination.

## Conclusion

This study was conducted to compare two attenuated IBD vaccines (strain V217 and strain M.B.) in terms of safety, antibody response to IBD vaccine, and antibody response to the Newcastle disease vaccine in commercial broilers. The V217-vaccinated group presented the highest NDV humoral response from 7 days dpv to the end of the study. The mean NDV antibody titer of the V217 group was significantly higher than the M.B.-vaccinated group at 14 dpv (p<0.05). In addition, the V217 strain-induced lower bursal lesions post-IBD vaccination and higher B:BW ratios at 7 and 21 dpv compared to the M.B. group. The higher B:BW ratio, lower bursal lesions, and higher ND seroconversion present in the V217 group indicated that the V217 strain-induced lower immunosuppressive effects compared to the M.B strain. The results of this study indicate that selecting IBD vaccine merits consideration, avoiding immunosuppressive effects induced by live IBD vaccination and the impact on the response to subsequent vaccines is important.

## Authors’ Contributions

TT: Conceptualization, methodology, investigation, data curation, writing – original draft and visualization, NC: Methodology, software and formal analysis, NCh: Conceptualization, methodology, investigation, resources, writing – review and editing, supervision, funding acquisition, and corresponding author. All authors read and approved the final manuscript.
